# Design of Controlled Release System for Paracetamol Based on Modified Lignin

**DOI:** 10.3390/polym11061059

**Published:** 2019-06-18

**Authors:** Mahboubeh Pishnamazi, Hamid Hafizi, Saeed Shirazian, Mario Culebras, Gavin M. Walker, Maurice N. Collins

**Affiliations:** 1 Department of Chemical Sciences, Bernal Institute, Synthesis and Solid State Pharmaceutical Centre (SSPC), University of Limerick, V94 T9PX Limerick, Ireland; Seyedeh.Pishnamazi@ul.ie (M.P.); Hamid.Hafizi@ul.ie (H.H.); Saeed.Shirazian@ul.ie (S.S.); gavin.walker@ul.ie (G.M.W.); 2 Stokes Laboratories, Bernal Institute, University of Limerick, V94 T9PX Limerick, Ireland; mario.culebrasrubio@ul.ie; 3 Health Research Institute, University of Limerick, V94 T9PX Limerick, Ireland

**Keywords:** lignin, drug release, paracetamol, disintegration

## Abstract

The influence of lignin modification on drug release and pH-dependent releasing behavior of oral solid dosage forms was investigated using three different formulations. The first formulation contains microcrystalline cellulose (MCC 101) as the excipient and paracetamol as the active pharmaceutical ingredient (API). The second formulation includes Alcell lignin and MCC 101 as the excipient and paracetamol, and the third formulation consists of carboxylated Alcell lignin, MCC 101 and paracetamol. Direct compaction was carried out in order to prepare the tablets. Lignin can be readily chemically modified due to the existence of different functional groups in its structure. The focus of this investigation is on lignin carboxylation and its influence on paracetamol control release behavior at varying pH. Results suggest that carboxylated lignin tablets had the highest drug release, which is linked to their faster disintegration and lower tablet hardness.

## 1. Introduction

Excipients play a significant role in the final product of pharmaceutical solid dosage forms. Variations in excipient properties influence tablet processability, hardness, disintegration and bioavailability [1,2,3]. Nowadays, many researchers have focused their investigations on using natural biopolymers [4] in tablet manufacturing due to their biocompatibility [5,6]; they are also cheap and widely available [7,8,9]. Lignin is a natural biopolymer with a number of beneficial properties including biodegradability and biocompatibility [10,11,12,13,14]. Recently, the use of lignin is increasing as a sustainable polymer for preparing carbon fibers [15], biofuels, bioplastics and controlled release carriers [16,17,18,19,20,21]. Due to the existence of different functional groups in the lignin structure such as phenolic, hydroxyl and carboxyl groups, lignin can be chemically modified to enhance drug delivery and to control drug release [22,23,24]. Figueiredo et al. functionalized Kraft lignin nanoparticles by carboxylation in order to improve drug delivery of poorly water-soluble anti-cancer drugs which were pH-sensitive [18]. Lievonen et al. modified softwood Kraft lignin using a dialysis technique to improve its drug delivery performance [25]. Furthermore, it has been recognized that pH-responsive drug carriers provide superior drug delivery characteristics due to their ability to increase the stability of the active pharmaceutical ingredient (API) molecules in the stomach and release the API in the intestine [26]. Li et al. investigated the release behavior of ibuprofen using lignin-based complex micelles. The results of release tests illustrated pH-dependent and controlled release properties due to ionization of the carboxyl groups in the lignin structure, with repulsive forces between the negatively-charged carboxyl groups of lignin and the API molecules, with higher solubility of the API at pH = 7.4 [27]. Chen et al. synthesized lignin-based pH-responsive nano-capsules to improve the controlled release of poorly water-soluble drugs by varying pH [28]. Duval et al. studied pH and light responsive behavior of controlled-release systems containing diazobenzen and modified softwood Kraft lignin [29]. Various investigations have been carried out on the effect of lignin-based polymeric nanoparticles (NPs) on the controlled release of pesticides [30,31].

Bulut et al. studied the controlled-release behavior of paracetamol using chitosan-graft-polyacrylamide microspheres via an emulsion crosslinking technique [32]. They utilized glutaraldehyde (GA) as a crosslinker to investigate its effect on the drug release rate. They mentioned the drug release rate was affected by some parameters such as the amount of GA, copolymer concentration and the composition of the drug and polymer. Their results illustrated that more controlled release of the drug occurred by increasing the GA amount and copolymer and decreasing the composition (paracetamol/polymer) ratio. Treenate et al. investigated the controlled release properties of paracetamol using a novel system composed of hydroxyethylacryl chitosan and sodium alginate in order to improve drug delivery for oral dosage forms [33]. Through improving drug water solubility, drug efficiency will be improved [34]. The current authors have evaluated the effect of lignin on the release rate of aspirin in oral dosage form, and indicated the higher release rate of drugs using lignin as an excipient in tablet formulation [9].

In this study, the effect of carboxylated lignin as an excipient on paracetamol release behavior was investigated. Lignin carboxylation was performed to enhance the carboxyl group content on the lignin surface in order to increase the interactions between the lignin and paracetamol functional groups and allow pH triggered release. To the best of our knowledge, no studies have reported the use of carboxylated lignin in paracetamol tablet manufacturing and its effect on the release. Three different formulations have been considered, one without lignin, one using pure lignin and one with carboxylated lignin. Paracetamol is utilized as a model drug in this research; it is a nonsteroidal anti-inflammatory [35]. Paracetamol is widely used as a pain relief drug as it has fast absorption within the small intestine of the human body [36,37]. Tablets were prepared by direct compaction and characterized using disintegration and dissolution tests. Modified lignin was verified using Fourier-transform infrared spectroscopy (FTIR). Drug release rates were measured using dissolution tests at pH 5.8 according to the United States Pharmacopeia (USP) [38]. In order to investigate the controlled release behavior of paracetamol, dissolution tests were carried out at acidic conditions (pH 1.2) and phosphate (pH 7.2) buffer solutions.

## 2. Experiments

### 2.1. Materials and Methods

Paracetamol (4-acetamidophenol, Phion) was used as a model API to prepare three different formulations. Microcrystalline cellulose (MCC SANAQ® 101 L USP/NF/EP) and Alcell lignin (Tecnaro, Ilsfeld, Germany) were used as excipients. More details on the lignin used in this study can be found elsewhere [2,15]. Table 1 shows the composition of the three formulations considered.

### 2.2. Lignin Modification

**Figure 1:**
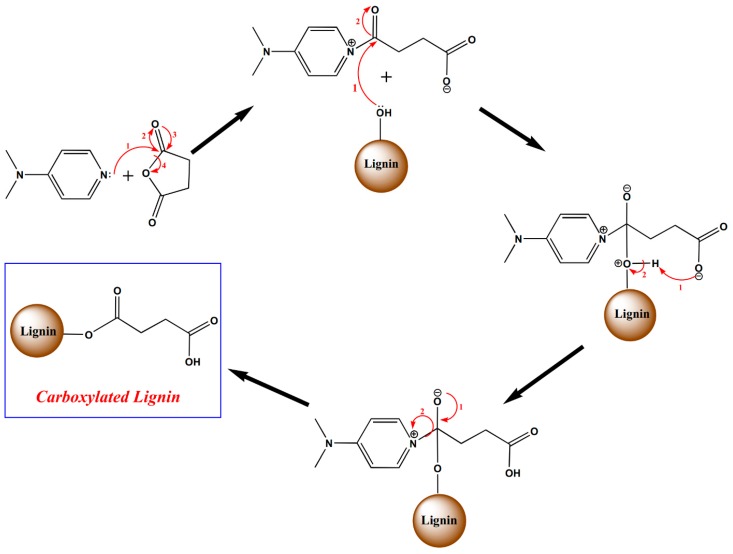
Mechanism of lignin carboxylation.

In order to allow conjugation reactions between lignin and paracetamol, lignin is functionalized with carboxylic acid groups. Synthesis of COOH–lignin involves a ring-opening reaction of succinic anhydride with 4-dimethylaminopyridine (DMAP). Lignin (2 g), succinic anhydride (2 g) and DMAP (400 mg) were added to 250 mL of tetrahydrofuran (THF) in a 500 mL round-bottom flask, followed by stirring for 48 h at room temperature [18]. The obtained carboxyl functionalized precipitate was filtered, and then, washed for 24 h using deionized water via the Soxhlet extraction system in order to remove the unreacted reagents. Finally, the modified lignin was placed in a freeze-dryer overnight. The proposed mechanism pathway [18] is presented in Figure 1.

### 2.3. Tablet Preparation

In order to prepare the tablets, a single-punch tablet press (Gamlen Tableting GTD-1 D series) was utilized. Each formulation (100 mg) was compacted to make each tablet in a 6 mm die. The tablet load was set at 400 kg, with a compaction rate of 180 mm/min.

### 2.4. Characterisation

Fourier-transform infrared spectroscopy (FTIR) measurements was carried out utilizing a Nicolet Nexus FTIR spectrometer between 450–4000 cm^−1^ equipped with an attenuated total reflectance accessory (ATR). A total of 60 scans were performed with a spectral resolution of 2 cm^−1^. Tablet hardness was measured using a tablet hardness tester (Pharma Test PTB311E). Pharma Test PTZ-DIST-Disintegration Test Instrument (Hainburg, Germany) was used to measure the tablet disintegration time. Deionized water (900 mL) was used to fill out the apparatus vessel and the peddle speed was kept constant at 100 rpm. The temperature of the vessel was adjusted to 37 °C. The tests were performed for the two formulations containing pure lignin and carboxylated lignin until the tablets completely disintegrated. A Pharma Test PTWS 120D 6-Station Tablet Dissolution Testing Instrument (Hainburg, Germany) was utilized to analyze the dissolution rate of the tablets. For the measurement of drug concentration, a Cary 60 UV Spectrophotometer (Agilent Technologies, Waldbronn, Germany) was used at a wavelength of 249 nm. All tests were carried out in triplicate. The calibration graph can be found in the Supplementary Information.

### 2.5. Dissolution Test Procedure

Phosphate buffer with pH = 5.8 (according to USP 23) was used as the dissolution medium [38,39]; 900 mL of medium was prepared to fill each dissolution vessel. The temperature of the medium chamber and the stirrer speed were kept constant at 37 ± 0.5 °C and 50 rpm, respectively. When running the dissolution test, first, the temperature should reach 37 °C. For each run, three vessels were utilized, and one tablet was considered for each vessel. Five milliliters of sample was withdrawn at 5 min, 10 min, 20 min, 30 min, 40 min, 50 min, 60 min and 120 min from each vessel and the same amount of medium was supplant, instantly. Afterwards, the samples were filtered by applying a Captiva Econofilter (PTFE membrane, 13 mm diameter, 0.2-µm pore size). Eventually, all samples were analyzed to measure the drug concentration using a Cary 60 UV Spectrophotometer at 249 nm wavelength, which was calibrated to the optimal wavelength. The cuvette type was 1/Q/10, quartz with pathway of 1 cm. In order to minimize the statistical error, all experiments were done in triplicate. For the dissolution tests of pH-responsive analysis, due to the evaluation of the controlled release behavior of paracetamol in the carboxylated lignin formulation, two different pHs were considered: phosphate buffer solution, pH = 7.2 (intestine environment) and acidic buffer solution (0.1 N HCL), pH = 1.2 (gastric environment) [40,41].

## 3. Results and Discussion

### 3.1. FTIR Characterization of Pure Lignin and Modified Lignin

**Figure 2:**
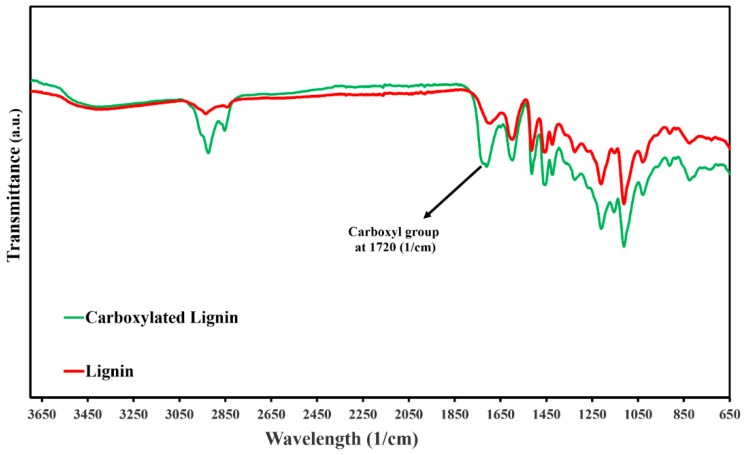
Fourier-transform infrared spectroscopy (FTIR) spectra of lignin (red) and carboxylated lignin (blue).

The FTIR spectra analysis was carried out to monitor the pure lignin structure and to characterize the chemical changes in the functional groups of the lignin structure during the carboxylation reactions. Figure 2 shows the spectra of pure lignin and functionalized lignin, which have similar peaks, such as C=O (carbonyl groups) at 1600 cm^−1^, –OH (hydroxyl groups) which are attributed to the phenol and alcohol in the region of 3600–3100 cm^−1^ and an aromatic ring region at 1425–1514 cm^−1^. Nevertheless, the hydrogen-bonded hydroxyl stretching band of carboxylic acid (2250–3600 cm^−1^) and the stretching vibrations of C=O of the unconjugated –COOH groups at 1720 cm^−1^ exhibit a stronger absorption bond than the pure lignin (unmodified), proving that grafting lignin with carboxylic acid groups has been successfully done.

### 3.2. Effect of Lignin Carboxylation on Tablet Disintegration Time

**Figure 3:**
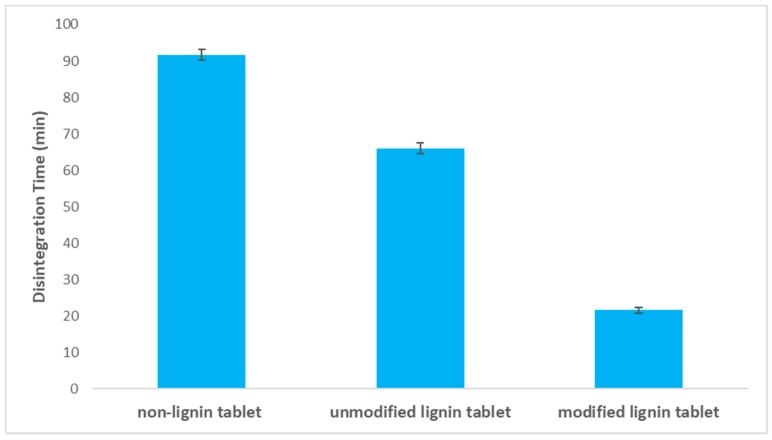
Disintegration time of tablets prepared containing pure lignin, modified lignin and no lignin.

**Figure 4:**
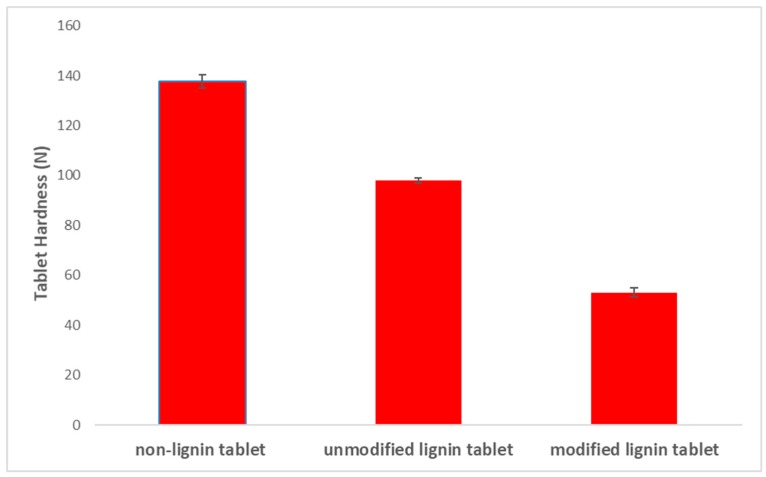
Hardness of tablets prepared containing pure lignin, modified lignin and no lignin.

Tablet disintegration time affects the tablet dissolution and can be used as a valuable test for solid oral dosage forms. Tablet hardness can influence tablet disintegration time, with higher hardness leading to longer disintegration times [42,43]. In order to study the effect of lignin carboxylation on the tablet disintegration time, a disintegration test was performed for the three different tablets (non-lignin, pure lignin and modified lignin). Figure 3 presents the disintegration time results in which a faster disintegration time for tablets containing modified lignin is obtained. Moreover, tablet hardness is measured using a hardness tester (Pharma Test, PTB) for three formulations, and the results show higher hardness for the formulation without lignin (Figure 4). Tablet hardness is affected by the physical properties of materials and the interaction of the drug with the excipient. The tableting method is the same for each formulation in order to mitigate its influence on tablet hardness. Generally, lower hardness equals to higher porosity; therefore, the lower hardness and higher porosity of the carboxylated lignin tablet is due presumably to the structural differences in lignin after modification. 

### 3.3. Effect of Lignin Carboxylation on Drug Release Rate

**Figure 5:**
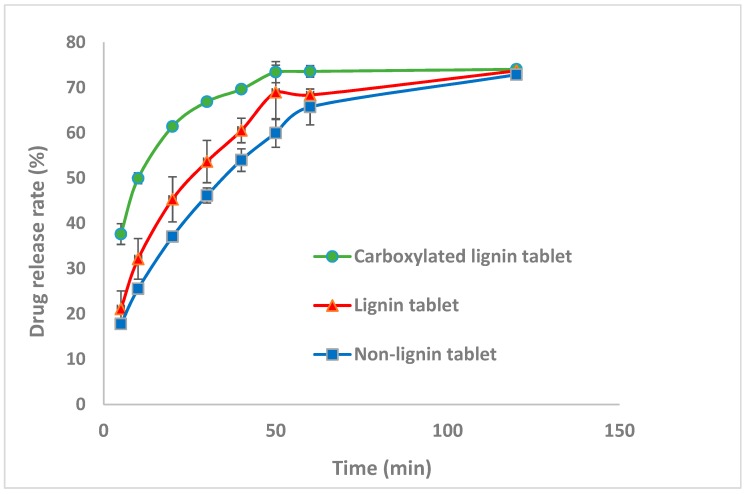
Drug release rate of paracetamol for the formulations at pH = 5.8.

Dissolution tests were performed to evaluate the effect of lignin and carboxylated lignin on the paracetamol tablet release rate. The three different formulations in Table 1 were considered to study paracetamol release rate in phosphate buffer solution at pH 5.8, according to the USP [38]. The release rate graphs of three different batches of paracetamol are displayed in Figure 5. Although the drug dissolution release is more dependent on the disintegration time, the formulation and material properties can influence the drug release rate. For this case, the graphs illustrate that the tablets containing functionalized lignin have the highest drug release rate and this correlates with the fastest disintegration time of these formulations and the lowest tablet hardness. 

Moreover, the prepared tablets containing pure lignin have a higher drug release rate compared to the formulation without lignin due to faster disintegration and lower tablet hardness [9]. Thus, lignin functionalization improved the release properties of directly compacted paracetamol tablets.

### 3.4. Controlled Release and pH-Responsive Behavior of Carboxylated Lignin

**Figure 6:**
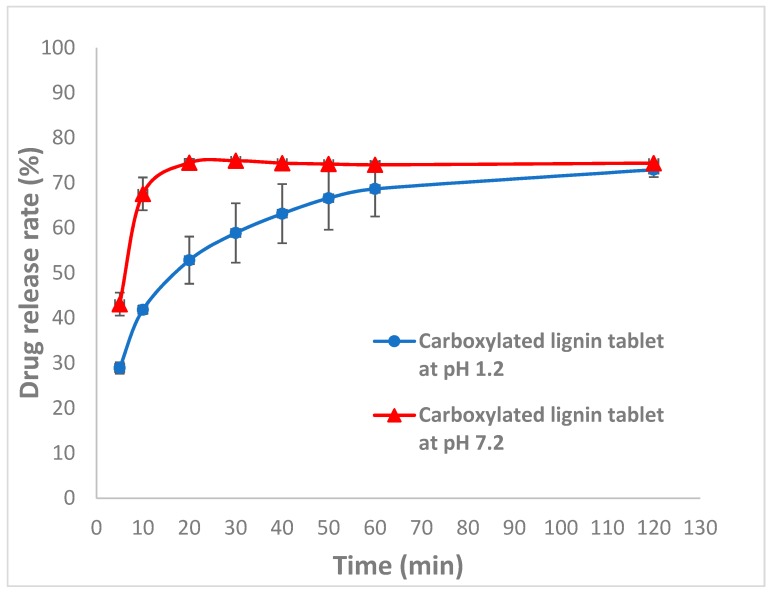
Drug release rate of carboxylated lignin in pH = 1.2 and pH = 7.2.

Drug release rate was studied in our previous work by adding lignin to the formulation [9]. The results revealed a higher drug release rate for the formulation containing lignin because of the amorphous structure of lignin and the interaction between lignin and the API, which resulted in an improvement in drug dissolution, which is the key factor in oral dosage development. Thus, for the present study, we have focused on drug controlled-release. The pH-responsive behavior of carboxylated lignin was investigated using dissolution tests in different media at various pH values: 0.1 M HCl solution (pH of 1.2, gastric environment) and phosphate buffer (pH 7.2, intestine environment) at 37 °C. The dissolution graphs in Figure 6 show a greater release rate of the drug in the buffer with pH = 7.2 [40]. Increasing the carboxyl groups results in an increase in drug release at pH = 7.2 compared to pH = 1.2. At pH = 1.2, the electrostatic repulsion between the lignin carboxyl groups decreases due to the protonation of carboxyl groups at lower pH values. However, at pH = 7.2, due to ionization of the carboxyl groups (pKa = 4.8) of modified lignin, the negatively-charged ions repel each other and presumably this leads to a swelling effect (similar to how hydrogels swell upon ionization [44]) and this results in higher release rates of the API. The results of this work reveal that lignin is a promising compound for use in controlled-release systems and also for enhancing the solubility of active pharmaceutical ingredients. This is the first time that modified lignin was used for the controlled-release of paracetamol. This could be very interesting from the point of view of lignin valorization since, at the moment, the market for high value applications is very limited. The use of lignin in the pharmaceutical industry can lead to the development of new value chains for lignin promoting circular bio-economy.

## 4. Conclusions

The aim of this study was to evaluate the pH-dependent release behaviors of modified lignin and the effect of lignin modification on the drug release rate. Lignin modification was conducted via carboxylation of lignin functional groups. In order to analyze the carboxyl groups in the structure of lignin and carboxylated lignin, an FTIR test was carried out and the results demonstrated a successful carboxylation. The dissolution results illustrate that there is a higher release rate of paracetamol from carboxylated lignin tablets, and this is attributed to the lower degree of interaction between lignin and the API due to the deprotonation of –COOH groups from modified lignin. Furthermore, the controlled release behavior of carboxylated lignin was evaluated at gastric pH of 1.2 and intestine pH of 7.2, and the release results showed the successful properties of controlled release. Additionally, the tablet disintegration tests showed a faster disintegration time for the carboxylated lignin tablets compared to pure lignin tablets due to the lower hardness of tablets with modified lignin. Thus, these investigations presented a successful use of carboxylated lignin natural biopolymer as an excipient in oral dosage forms for desired drug controlled-release. 

## Figures and Tables

**Table 1:** Various formulations used in this study.

Material	Formulations		
	A	B	C
Paracetamol (wt %)	20	20	20
Alcell lignin (wt %)	0	10	0
Modified Alcell lignin (wt %)	0	0	10
MCC 101 (wt %)	80	70	70

MCC = microcrystalline cellulose.

## Supplementary Materials

The following are available online at https://www.mdpi.com/2073-4360/11/6/1059/s1.
