# Supplementary material for: Design of Controlled Release System for Paracetamol Based on Modified Lignin
Source: Polymers (Basel). 2019 Jun 18;11(6):1059. doi: 10.3390/polym11061059 (PMC6630591; doi:10.3390/polym11061059)
Supplement: Supplementary file 1 [file polymers-11-01059-s001.pdf]

polymers

Supplementary File

Design of Controlled Release System for Paracetamol Based on Modified Lignin

Mahboubeh Pishnamazi1, Hamid Hafizi1, Saeed Shirazian1, Mario Culebras2, Gavin M. Walker1, Maurice N. Collins2,3*
1 Department of Chemical Sciences, Bernal Institute, Synthesis and Solid State Pharmaceutical Centre (SSPC), University of Limerick, Limerick, Ireland
2 Stokes Laboratories, Bernal Institute, University of Limerick, Limerick, Ireland
3 Health Research Institute, University of Limerick, Limerick, Ireland.
* Corresponding author, E-mail: Maurice.Collins@ul.ie

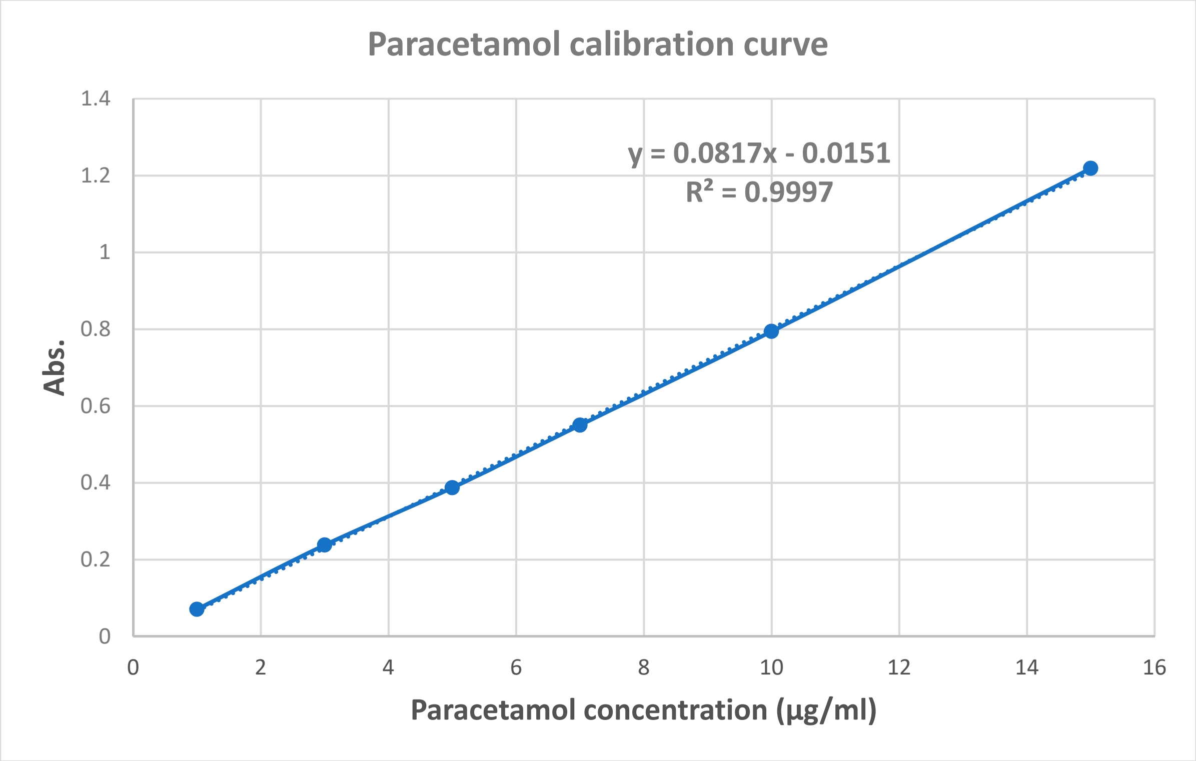

Calibration curve

Explanation of Lignin Modification Mechanism

In the first step, the lone pair of nitrogen in DMAP, as nucleophiles, attack at the carbonyl group of succinic anhydrous and ring opening occurs. Afterwards, hydroxyl groups of lignin react with the intermediate, which was formed in the first step. Following this, there is a proton transfer between two oxygen atoms (neutralization) and in the last step, carbonyl (C=O) forms with the DMAP acting as a cleaving group, resulting in a carboxylated lignin.
